# ID2 Inhibits Bladder Cancer Progression and Metastasis *via* PI3K/AKT Signaling Pathway

**DOI:** 10.3389/fcell.2021.738364

**Published:** 2021-10-22

**Authors:** Weipu Mao, Keyi Wang, Si Sun, Jianping Wu, Ming Chen, Jiang Geng, Ming Luo

**Affiliations:** ^1^Department of Urology, Shidong Hospital of Yangpu District, Shanghai, China; ^2^Department of Urology, Affiliated Zhongda Hospital of Southeast University, Nanjing, China; ^3^Surgical Research Center, Institute of Urology, Southeast University Medical School, Nanjing, China; ^4^Department of Urology, School of Medicine, Shanghai Tenth People’s Hospital, Tongji University, Shanghai, China

**Keywords:** bladder cancer, ID2, PI3K/AKT signaling pathway, progression, metastasis

## Abstract

**Background:** Inhibitors of DNA-binding (ID) proteins are important regulators of cell proliferation and differentiation. The aim of this study was to evaluated the role of ID proteins in bladder cancer (BCa) and related molecular mechanisms.

**Methods:** The TCGA database was analyzed for the expression and clinical significance of ID proteins. The expression of ID2 was determined by qRT-PCR, immunohistochemical staining and western blot. The role of ID2 was determined by CCK-8, colony formation, wound healing, transwell and xenograft tumor assays, and the potential mechanism of ID2 in BCa was investigated by RNA sequencing.

**Results:** ID2 expression was significantly downregulated in TCGA database and clinical samples, and high ID2 expression was associated with low-grade tumor staging and correlated with better overall survival, disease specific survival (DSS) and progress free interval (PFI). *In vivo* and *in vitro* experiments showed that knockdown of ID2 promoted proliferation, migration, invasion and metastasis of BCa cells, while overexpression of ID2 significantly inhibited cell proliferation, migration, invasion and metastasis. Mechanistically, ID2 acts as a tumor suppressor through PI3K/AKT signaling pathway to inhibit the progression and metastasis of BCa.

**Conclusion:** Our results suggest that ID2 exerts tumor suppressive effects in BCa through PI3K/AKT signaling pathway, and altered ID2 expression can be used as a biomarker of BCa progression and metastasis.

## Background

Bladder cancer (BCa) is one of the tumors with high morbidity and mortality in the urinary system. In 2020, BCa ranked 12th in incidence and 13th in mortality among all malignant tumors (Sung et al., 2021). The latest statistical results from China Tumor Registry show that the incidence rate of BCa in China was 8.0/100,000 and the mortality rate was 3.3/100,000 in 2015 (Zhang et al., 2020). BCa can be divided into non-muscle invasive bladder cancer (NMIBC) and muscle invasive bladder cancer (MIBC) based on muscle infiltration (Mao et al., 2019). NMIBC accounts for approximately more than 75% of BCa and the preferred treatment is transurethral resection of bladder tumour (TURBt) (Dy et al., 2017). Although NMIBC generally has a good prognosis, it has a high recurrence rate and about 10–15% of patients will progress to MIBC in the course of treatment (Ranzi et al., 2017). MIBC is highly aggressive and prone to metastasis, and patients have a poor prognosis (Robertson et al., 2017). After the progression of NMIBC to MIBC, radical cystectomy is generally chosen as the treatment modality (Witjes et al., 2021). However, the cost of surgery is significantly higher, the quality of life after surgery is significantly lower, and the burden on family and society is significantly higher (Garcia-Donas et al., 2017). Therefore, it is particularly important to explore the mechanisms of BCa progression, discover new biomarkers and identify effective therapeutic targets for BCa.

Inhibitors of DNA-binding (ID) proteins are important regulators of cell proliferation and differentiation. ID family proteins are a class of proteins that contain the basic Helix-Loop-Helix (bHLH) structural domain while lacking a DNA binding sites, which belong to the HLH family and are negative regulators of the basic HLH transcription factors (Benezra et al., 1990). ID proteins can inhibit cell differentiation and can induce cell proliferation by regulating different cell cycle regulators to enhance tissue invasiveness and angiogenesis of tumor cells (Perk et al., 2005). ID proteins are expressed in many tissues and organs, but their expression is somewhat variable in different developmental stages and tissues (Massari and Murre, 2000). An increasing number of studies have confirmed that abnormal expression of ID proteins is not only involved in tumor development, but also closely related to tumor invasion and metastasis formation (Volpert et al., 2002; Fong et al., 2004). However, the predictive value of ID proteins for BCa and their possible molecular mechanisms have not been elucidated.

In this study, we examined the expression of ID proteins (ID1, ID2, ID3, and ID4) in BCa tissues and found that ID2 expression was downregulated and associated with tumor stage and survival. Further analysis showed that ID2 inhibited BCa proliferation, migration, invasion and metastasis through the PI3K/AKT signaling pathway. Taken together, our study suggests that ID2 may be a potential therapeutic target for BCa.

## Materials and Methods

### Clinical Specimens

Twenty-five pairs of BCa tumor tissues and corresponding adjacent normal tissues were collected from BCa patients who underwent radical cystectomy between January 2016 and December 2016 at Shanghai Tenth People’s Hospital (Shanghai, China). None of the patients in this study received any radiotherapy or chemotherapy before the surgery. Pathology of all BCa patients was confirmed by hospital pathologists, and pathological staging was determined according to the American Joint Committee on Cancer TNM staging system (7th edition). The study was evaluated and approved by the Ethics Committee of the Shanghai Tenth People’s Hospital (SHSY-IEC-4.1/19-120/01) and was conducted in accordance with the relevant regulations. All patients or their relatives had written informed consent.

### The Cancer Genome Atlas Database

ID1, ID2, ID3, and ID4 expression in BCa and related clinical data are available from the Cancer Genomics Browser. In brief, we downloaded RNA-seq data and clinical information from the TCGA database for 433 BCa projects, including 19 cases with matched normal tissues. The downloaded data were used in transcripts per million (TPM) format for analysis. In addition, we downloaded RNA-seq data in TPM format from the TCGA and Genotype-Tissue Expression (GTEx) databases.

### Cell Lines and Culture

The immortalized human normal bladder epithelial cell line SV-HUC-1 and human BCa cell lines UMUC3, 5637, T24, and EJ were purchased from the Cell Bank of the Chinese Academy of Sciences (Shanghai, China). UMUC3, 5637, T24, and EJ cells were cultured in RPMI-1640 medium (Gibco; Thermo Fisher Scientific, United States) and SV-HUC-1 cells were maintained in F12K medium (Sigma-Aldrich; Merck KGaA, Germany). All cell cultures were supplemented with 10% fetal bovine serum (FBS, Gibco; Thermo Fisher Scientific, United States) and 1% penicillin/streptomycin (Gibco; Thermo Fisher Scientific, United States) and cultured at 37°C in a humidified incubator containing 5% CO_2_.

### Cell Transfection

Small interfering RNAs specifically targeting ID2 (si-ID2: GGACTCGCATCCCACTATT) and negative control siRNA (Control) were purchased from RiboBio (Guangzhou, China). Transient transfection was performed at 30–50% cell confluence using Lipofectamine 3000 (Thermo Fisher Scientific, United States). ID2 knockdown lentivirus (sh-ID2) carrying si-ID2, Control lentivirus and ID2 overexpression lentivirus (OE-ID2) carrying si-ID2, Control, or ID2 sequences, respectively, and the lentivirus were constructed by BioLink (Shanghai, China). sh-ID2, Control, and OE-ID2 stable transfer cell lines were generated by lentivirus transfection.

### RNA Sequencing Analysis

To find ID2-associated downstream pathways, we performed RNA sequencing analysis on EJ cell line transfected with OE-ID2, sh-ID2, and control lentivirus. mRNA expression analysis was performed on Agilent’s whole human genome microarray 4 × 44 K v2 (026652) with monochrome hybridization, including probes for 34184 human mRNA transcripts. RNA sequencing was performed according to the previously described procedure (Luo et al., 2019). Sample preparation and microarray hybridization were performed according to the standard protocol of Arraystar (Majorbio, Shanghai, China). In addition, further Gene Ontology (GO) and Kyoto Encyclopedia of Genes and Genomes (KEGG) enrichment analysis was used to screen for signaling pathways.

### RNA Extraction and Quantitative Real-Time Polymerase Chain Reaction

Total RNA was extracted from cells or human tissue using Trizol reagent (TaKaRa, China) according to the manufacturer’s instructions. Reverse transcription was performed using cDNA kits (R312, Vazyme Biotech, Nanjing, China) to synthesize cDNA. CT values were detected by qRT-PCR using SYBR Green PCR kit (Q141, Vazyme Biotech, Nanjing, China) and the ABI Prism 7500 sequence detection system (Applied Biosystems, United States). Primers for ID2 are listed below: ID2-F 5′ TCAGCACTTAAAAGATTCCGTG 3′; ID2-R 5′ GA CAGCAAAGCACTGTGTGG 3′; PI3K-F 5′ ATCAACAGCCAA CAAATACC 3′; PI3K-R 5′ TTCTTATCACCGTCACCCT 3′; Akt-F 5′ ATCAACAGCCAACAAATACC 3′; Akt-R 5′ TTCTTA TCACCGTCACCCT 3′; SGK3-F 5′ TGGGGCTGTTCTGTA TGAAATGCTG 3′; SGK3-R 5′ TGGACCAGGCTGTAAGA CTCACTC 3′; GAPDH-F 5′ AACGGATTTGGTCGTATTG 3′; GAPDH-R 5′ GGAAGATGGTGATGGGATT 3′. The relative expression of ID2 was calculated using the 2^–Δ^ ^Δ^ ^Ct^ method and GAPDH was used as internal standards.

### Cell Counting Kit-8 Assays

Transfected EJ and UNUC3 cells were seeded at a density of 2,000 cells per well into 96-well plates (Corning, United States). After seeding for 12, 24, 48, 72, and 96 h, 100 μl of serum-free medium and 10 μl of CCK8 solution (Yeasen, Shanghai, China) were added to each well and incubated at 37°C in the dark for 2 h, and optical density (OD) was detected at 450 nm.

### Colony Formation Assays

Transfected cells were plated into 6-well plates (Corning, United States) at a density of 500 per well and cultured with complete medium for approximately 2 weeks. The culture was terminated when clones were visible to the naked eye in the culture dish. 2 weeks later, colonies were fixed using formaldehyde and then stained with 0.1% crystalline violet (Vicmed, China). These colonies were subsequently photographed and counted.

### Wound Healing Assay

The transfected cells were seeded into 6-well plates (Corning, United States). When cells were reconnected and reached 80% confluence, cell monolayers were scratched with a 200 μL pipette. Subsequently, cells were washed with PBS to remove cell debris and medium containing 2% FBS was added. At 0, 12, and 24 h after injury, images were acquired at the same locations and wound area was calculated using ImageJ software (NIH, United States).

### Cell Apoptosis Assay

Transfected cells were grown into 6-well plates (Corning, United States), and all cells in the medium and adherent to the wall were collected when the cells grew to 80% confluence. Cells were washed twice with cold 1 × PBS, Annexin V (BD Biosciences, United States) binding buffer was added, and then stained for 15 min at room temperature in the dark using fluorescein isothiocyanate (FITC) and propidium iodide (PI). Finally, the apoptosis rate was detected using BD FACS Calibur (Beckman Coulter, CA, United States).

### Transwell Migration and Invasion Assays

Both cell migration and invasion ability assays were performed using Transwell chambers (8 μm pore size, Corning, United States). The upper chamber was not covered with Matrigel (BD Biosciences, United States) for the migration assay and 100 μl Matrigel for the invasion assay. Specifically, transfected cells (5 × 10^4^) were inoculated in the upper chamber and culture medium containing 10% FBS was placed in the lower chamber. After 12–24 h incubation, the invading and migrating cells were fixed, stained with 0.1% crystalline violet (Vicmed, China), photographed and counted using an inverted microscope (Leica Microsystems, Germany).

### Western Blot Analysis

Cells were lysed with RIPA buffer (Beyotime, China) containing protease inhibitors on ice and proteins were extracted, and protein concentrations were determined using BCA protein assay kit (Thermo Fisher Scientific, United States). Protein lysates (50 μg/lane) were separated by 10% sodium dodecyl sulfate-polyacrylamide gels (SDS-PAGE) electrophoresis and transferred to polyvinylidene fluoride membranes (Merck, United States). The membranes were subsequently blocked with 5% skim milk for 1 h and incubated with primary antibodies (Supplementary Table 1) overnight at 4°C. Subsequently, the membranes were incubated with secondary mouse or rabbit antibodies at room temperature for 1 h. After washing three times with PBST, the signals were observed using a Tanon (Shanghai, China) chemiluminescence image analysis system.

### Xenograft Tumor Models

Forty 4-week male M-NSG mice were purchased from Model Biological Center Inc., (Shanghai, China), and the mice were randomly divided into 8 groups (*n* = 5 per group).

Subcutaneous xenograft tumor model: UMUC3 cells stably transfected with sh-ID2 or Control and EJ cells stably transfected with Control or OE-ID2 were collected and resuspended in saline. 100 μl of 5 × 10^7^ density cells were mixed with 100 μl Matrigel (BD, United States) and injected subcutaneously into the mice. The length and width of the tumors were measured weekly and the tumor volume was calculated using the formula: volume (mm^3^) = 0.5 × width^2^ × length. Mice were sacrificed after 4 weeks, the subcutaneously transplanted tumors were excised, and the weight of each tumor was recorded. A portion of the tumor tissue was fixed in 10% buffered formalin and subjected to subsequent analysis.

*In vivo* lung metastasis model: Cell lines were constructed as described above in “Cell transfection.” UMUC3 cells stably transfected with sh-ID2 or Control and EJ cells stably transfected with Control or OE-ID2 were collected and resuspended in saline. 1 × 10^6^ cells were injected from the tail vein of mice in a volume of 200 μl. Mice were executed after 4 weeks, lung tissue was excised, and fixed in 10% buffered formalin to observe the number of metastatic nodules in the lungs was observed using the *in vivo* imaging system (IVIS) imaging system (Calipers, Hopkinton, United States) for observation and subsequent analysis.

### Haematoxylin and Eosin and Immunohistochemical Staining

Mice lung tissues were embedded in paraffin and sectioned at a thickness of 5μm, followed by H&E staining. Mice tumor tissues were fixed with 4% paraformaldehyde and embedded in paraffin after dehydration through ethanol solution. IHC was performed according to the previously described protocol (Mao et al., 2020), followed by recording images with a microscope (Leica Microsystems, Germany).

### Statistical Analysis

The relationship between ID2 expression and various clinicopathological variables was examined using the chi-square test. SPSS 20.0 software (IBM, United States), GraphPad Prism 8.3 software (San Diego, United States), and R-Studio software (Boston, United States) were used for all statistical analyses. For all studies, *P*< 0.05 between groups were considered statistically significant.

## Results

### ID2 Expression Correlates With Clinical Characteristics and Survival Rate in the Cancer Genome Atlas Database

**FIGURE 1:**
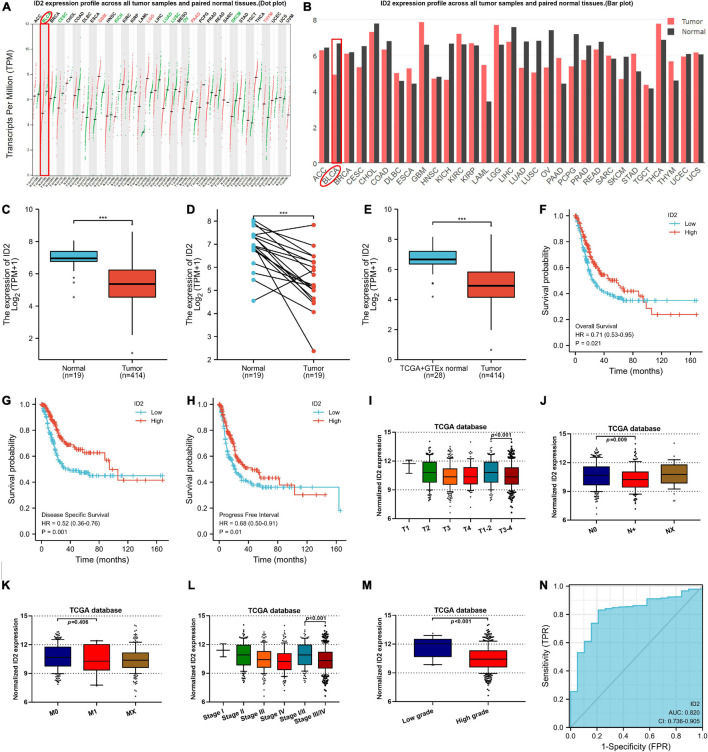
ID2 expression correlates with clinical characteristics and survival rate in the TCGA database. **(A,B)** ID2 expression profile across all tumor samples and paired normal tissues. **(C)** The difference expression of ID2 in BCa tissues and adjacent normal tissues. **(D)** The difference expression of ID2 in BCa tissues and paired normal tissues. **(E)** The difference expression of ID2 in normal tissues of GTEx combined with TCGA and BCa tissues of TCGA. **(F–H)** Overall survival **(F)**, disease-specific survival **(G)**, and progress free interval **(H)** curve of BCa patients with low (*n* = 207) and high (*n* = 207) ID2 expression. **(I–M)** Relative expression levels of ID2 in TCGA database with T stage **(I)**, N stage **(J)**, M stage **(K)**, pathological stage **(L)**, tumor grade **(M)**. **(N)** ROC curve showed the efficiency of ID2 expression level to distinguishing BCa tissue from non-tumor tissue (***p < 0.001).

To explore the expression of ID1, ID2, ID3, and ID4 in BCa, we first detected and analyzed their expression in the TCGA database. We analyzed their expression in 414 BCa tissues and 19 normal tissues, 19 BCa tissues and matched normal tissues, and found that ID2, ID3, and ID4 were downregulated in BCa tissues (Figures 1A–D and Supplementary Figures 1H–J, O–Q), while ID1 expression was not different (Supplementary Figures 1A–C). In addition, we also compared the expression of ID1, ID2, ID3, and ID4 in 28 normal samples from the GTEx combined TCGA database and 414 BCa samples from the TCGA database, and similarly found low expression of ID2, ID3 and ID4 (Figure 1E and Supplementary Figures 1K,R) and no difference in ID1 expression (Supplementary Figure 1D). Further survival analysis revealed that ID1 and ID2 expression were associated with overall survival (OS) (Figure 1F and Supplementary Figure 1L), disease specific survival (DSS) (Figure 1G and Supplementary Figure 1M), and progress free interval (PFI) (Figure 1H and Supplementary Figure 1N), while ID3 expression was not associated with survival (Supplementary Figures 1E–G,S–U). Subsequently, we examined the relationship between ID2 and clinical variables and found that ID2 expression correlated with TNM stage (Figures 1I–K), pathological stage (Figure 1L) and grade (Figure 1M) in TCGA patients. In addition, the chi-squared assay showed that ID2 expression correlated with T-stage, pathological stage and grade (Table 1). Moreover, receiver operating characteristic (ROC) curves were used to analyze the effectiveness of ID2 expression levels in distinguishing BCa tissue from normal tissue. The area under curve (AUC) of ID2 was 0.820 (Figure 1N), indicating that ID2 can be used as an ideal biomarker to distinguish BCa from normal tissue.

**TABLE 1:** The relationship between the expression of ID2 and various clinicopathological variables in the TCGA database.

**Characteristics**	**Total**	**ID2 expression**		***P*-value**
		**Low**	**High**	
Total	414	207	207	
Age (years)				0.921
≤70	234	116 (28.0%)	118 (28.5%)	
>70	180	91 (22.0%)	89 (21.5%)	
Sex				0.264
Female	109	60 (14.5%)	49 (11.8%)	
Male	305	147 (35.5%)	158 (38.2%)	
T-stage				**0.014**
T1	5	0 (0.0%)	5 (1.3%)	
T2	119	52 (13.7%)	67 (17.6%)	
T3	196	110 (28.9%)	86 (22.6%)	
T4	60	33 (8.7%)	27 (7.1%)	
N-stage				0.160
N0	239	112 (30.3%)	127 (34.3%)	
N1	46	27 (7.3%)	19 (5.1%)	
N2	77	46 (12.4%)	31 (8.4%)	
N3	8	4 (1.1%)	4 (1.1%)	
M-stage				0.657
M0	203	97 (45.5%)	105 (49.3%)	
M1	11	4 (1.9%)	7 (3.3%)	
Pathological stage				**0.004**
Stage I	4	0 (0.0%)	4 (1.0%)	
Stage II	130	53 (12.9%)	77 (18.7%)	
Stage III	142	74 (18.0%)	68 (16.5%)	
Stage IV	136	80 (19.4%)	56 (13.6%)	
Grade				**0.023**
High grade	390	202 (49.1%)	188 (45.7%)	
Low grade	21	5 (1.2%)	16 (3.9%)	

*P < 0.05 are shown in bold.*

### ID2 Is Down-Regulated in Bladder Cancer Clinical Sample

**FIGURE 2:**
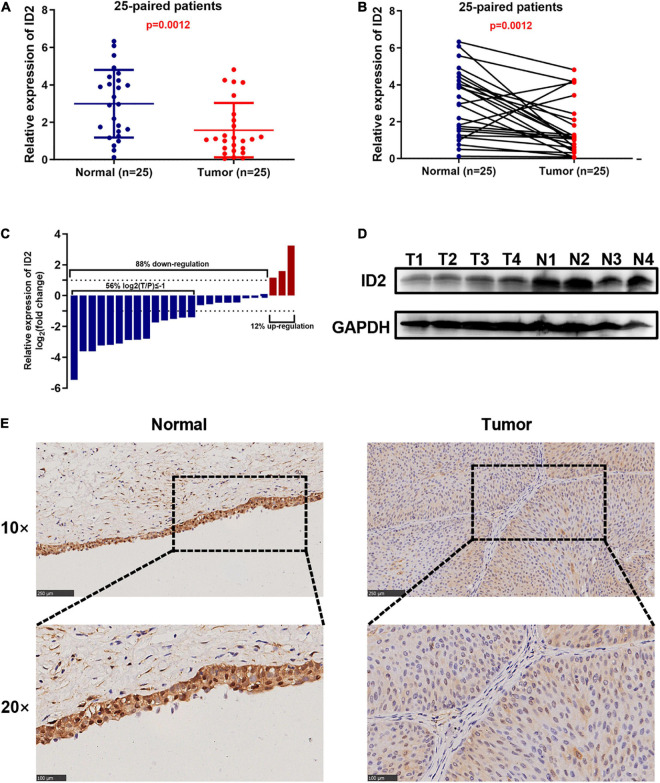
ID2 is down-regulated in BCa clinical sample. **(A–D)** qRT-PCR **(A–C)** and western blot **(D)** analysis of ID2 expression levels in BCa tissues and paired normal tissues. **(E)** Representative IHC images showing ID2 staining in normal tissues (N) and BCa tumor (T) sections.

To further validate the conclusion that ID2 is downregulated expression in the TCGA database, we examined the ID2 mRNA expression levels in 25 paired BCa tissue samples by qRT-PCR. The results revealed that ID2 expression was low expression in tumor tissues (Figures 2A–C) and similar results were observed by western blotting (Figure 2D). The clinical and pathological characteristics of our patients are shown in Table 2, and we found that ID2 expression was associated with T-stage and M-stage. In addition, IHC results also showed that ID2 expression was significantly down-regulated in tumor tissues (Figure 2E).

**TABLE 2:** The relationship between the expression of ID2 and various clinicopathological variables in our center.

**Characteristics**	**Total**	**ID2 expression**		***P*-value**
		**Low**	**High**	
Total	25	12	13	
Age (years)				0.073
<70	13	4 (33.3)	9 (69.2)	
≥70	12	8 (66.7)	4 (30.8)	
Sex				0.035
Male	18	11 (91.7)	7 (53.8)	
Female	7	1 (8.3)	6 (46.2)	
T-stage				**0.019**
T1-T2	18	6 (50.0)	12 (92.3)	
T3-T4	7	6 (50.0)	1 (7.7)	
N-stage				0.114
N0	17	10 (83.3)	7 (53.8)	
N1/N2	8	2 (16.7)	6 (46.2)	
M-stage				**0.047**
M0	19	7 (58.3)	12 (92.3)	
M1	6	5 (41.7)	1 (7.7)	
Tumor size (cm)				0.543
<3	13	7 (58.3)	6 (46.2)	
≥3	12	5 (41.7)	7 (53.8)	

*P < 0.05 are shown in bold.*

### ID2 Inhibits Proliferation, Migration, and Invasion of Bladder Cancer Cells *in vitro*

**FIGURE 3:**
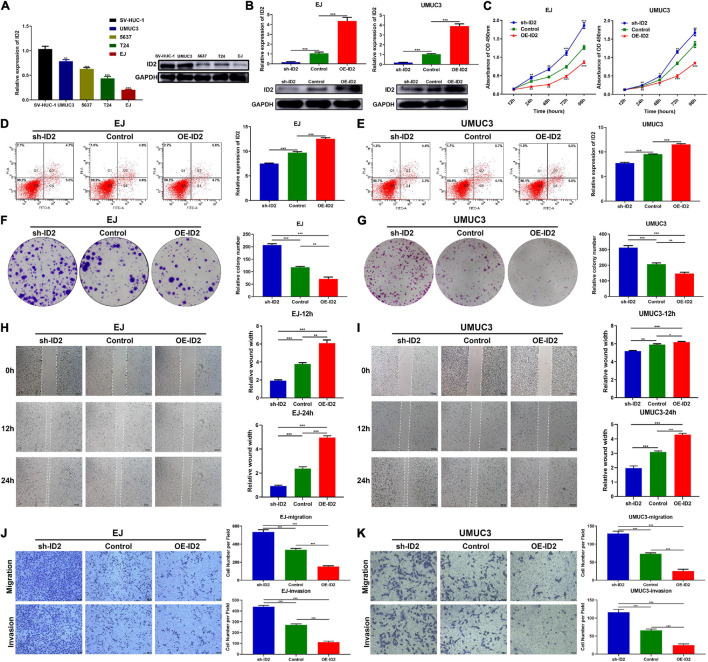
ID2 inhibits proliferation, migration and invasion of BCa cells *in vitro*. **(A)** Relative expression of ID2 in SV-HUC-1 cell and BCa cell lines. **(B)** Expression of ID2 was confirmed by qRT-PCR in BCa cell lines EJ and UMUC3 transfected with sh-ID2, Control or OE-ID2. **(C)** CCK8 assay of cell proliferation capacity of EJ and UMUC3 cells after transfection with sh-ID2, Control, or OE-ID2. **(D,E)** Cell apoptosis assay were performed in EJ **(D)** and UMUC3 **(E)** cells after transfection with sh-ID2, Control or OE-ID2. **(F,G)** Colony formation assays were performed in EJ **(F)** and UMUC3 **(G)** cells after transfection with sh-ID2, Control, or OE-ID2. **(H,I)** Wound healing assays were performed in EJ **(H)** and UMUC3 **(I)** cells after transfection with sh-ID2, Control, or OE-ID2. **(J,K)** Transwell assays were performed in EJ **(J)** and UMUC3 **(K)** cells after transfection with sh-ID2, Control, or OE-ID2 (*p < 0.05, **p < 0.01, ***p < 0.001).

We first examined the expression of ID2 in BCa cell lines, and the results showed that ID2 was lowly expressed at both the mRNA level and protein level (Figure 3A). To investigate the biological role of ID2 in BCa cells, we designed siRNA against ID2 (si-ID2) and an overexpression plasmid (OE-ID2), which were transfected into EJ and UMUC3 cells. qRT-PCR showed that si-ID2 decreased ID2 expression, while transfection with OE-ID2 could upregulated ID2 expression levels (Figure 3B). Apoptosis assays showed that inhibition of ID2 expression inhibited apoptosis and overexpression promoted apoptosis in EJ and UMUC3 cells (Figures 3D,E). CCK-8 and colony formation assays showed that inhibition of ID2 significantly enhanced proliferation of EJ and UMUC3 cells, while overexpression of ID2 had the opposite effect (Figures 3C,F,G). In addition, wound healing and Transwell assays also showed that silencing ID2 enhanced the migration and invasion ability of EJ and UMUC3 cells, while overexpression of ID2 decreased the migration and invasion ability of the cells (Figures 3H–K).

### ID2 Suppressed the Growth and Metastasis of Bladder Cancer Cells *in vivo*

**FIGURE 4:**
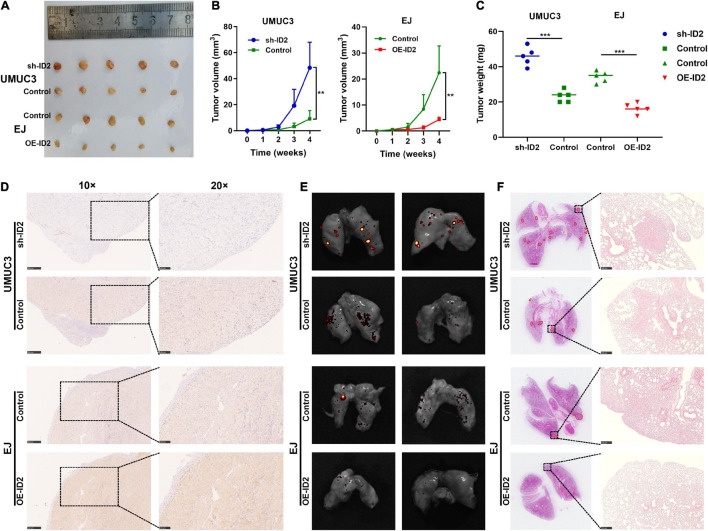
ID2 suppressed the growth and metastasis of BCa cells *in vivo*. **(A)** Representative images of xenograft tumors in nude mice. **(B)** The growth curves of xenografts. **(C)** The tumor weight of xenografts. **(D)** IHC assay demonstrated the level of ID2 in pairs of tumors. **(E,F)** Representative *in vitro* imaging **(E)** and H&E images **(F)** of lung tissue sections (**p < 0.01, ***p < 0.001).

To assess the effect of ID2 on BCa growth and metastasis *in vivo*, we designed subcutaneous xenograft tumor models and lung metastasis models. UMUC3 cells stably transfected with sh-ID2 and Control, and EJ cells stably transfected with Control and OE-ID2 were injected subcutaneously into M-NSG mice to construct xenograft tumor models. As expected, after 4 weeks, the tumor volume and weight in the sh-ID2 group were higher than those in the control group, and the tumor volume and weight in the OE-ID2 group were smaller than those in the control group (Figures 4A–C). IHC assays showed that the expression of ID2 was significantly up-regulated in the OE-ID2 group and down-regulated in the sh-ID2 group compared to the Control group (Figure 4D).

In addition, the above-mentioned stable transfer cells were injected tail vein into mice. *In vitro* imaging and H&E staining results showed that the sh-ID2 group had more metastatic foci in the lungs of the mice, while the mice in the OE-ID2 group had essentially no metastatic foci (Figures 4E,F).

### ID2 Regulated Bladder Cancer Progression via PI3K/AKT Signaling Pathway

**FIGURE 5:**
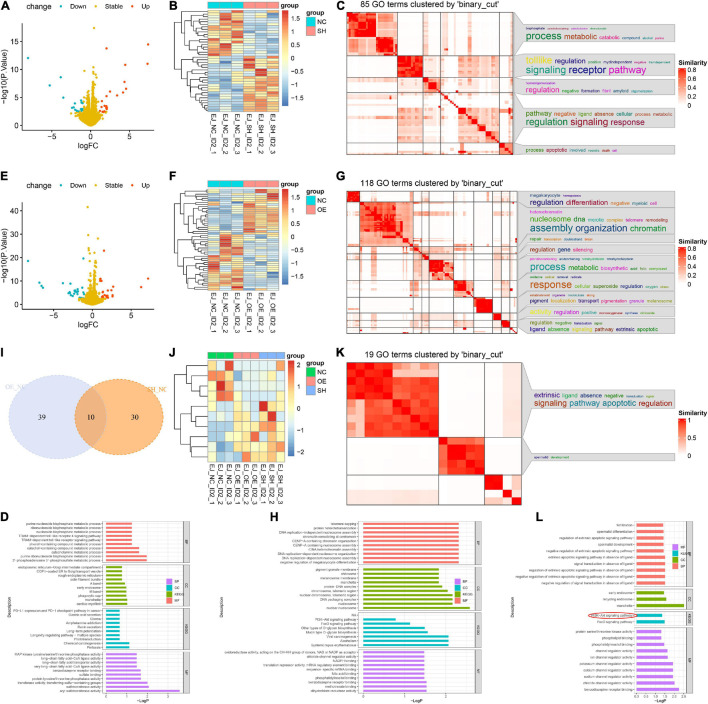
ID2 regulated BCa progression via PI3K/AKT signaling pathway. **(A,B)** Volcano plot **(A)** and heatmap **(B)** of RNA-Seq analysis of NC-ID2 and sh-ID2 cells. **(C,D)** GO terms **(C)** and KEGG analysis **(D)** to detect differentially expressed genes. **(E,F)** Volcano plot **(E)** and heatmap **(F)** of RNA-Seq analysis of NC-ID2 and OE-ID2 cells. **(G,H)** GO terms **(G)** and KEGG analysis **(H)** to detect differentially expressed genes. **(I)** Venn diagrams show the number of genes with changes in NC-ID2 and sh-ID2, NC-ID2, and OE-ID2. **(J)** Heatmap of RNA-Seq analysis of sh-ID2, NC-ID2, and OE-ID2 cells. **(K,L)** GO terms **(K)** and KEGG analysis **(L)** to detect differentially expressed genes.

To decipher the downstream-related pathways of ID2 in BCa progression, we constructed sh-ID2, NC-ID2, and OE-ID2 EJ stable transduction cell lines and sequenced the three cell lines at the transcriptional level (Supplementary Table 2). We first analyzed sh-ID2 and NC-ID2 and identified 18 down-regulated differentially expressed genes (DEGs) and 22 up-regulated DEGs (Figures 5A–D). Subsequent analysis of NC-ID2 and OE-ID2 revealed 22 genes with high expression and 27 genes with low expression (Figures 5E–H). Further analysis showed that 10 genes were co-variant (Figures 5I,J). We performed GO analysis and KEGG analysis on 10 genes and found that PI3K/AKT signaling pathway was significantly enriched (Figures 5K,L and Supplementary Table 3).

**FIGURE 6:**
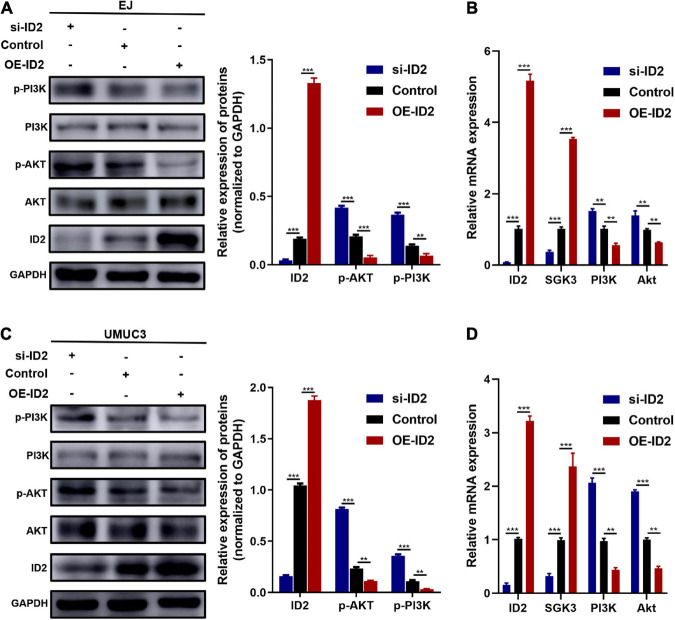
Protein and mRNA changes in EJ and UMUC3 cells after transfection with si-ID2, NC-ID2 and OE-ID2. **(A,B)** Protein and mRNA changes in EJ cells. **(C,D)** Protein and mRNA changes in UMUC3 cell (**p < 0.01, ***p < 0.001).

Subsequently, we examined the expression changes of PI3K/AKT signaling pathway related proteins (AKT and PI3K). Western blotting results showed that transfection of sh-ID2 could lead to decreased protein levels of ID2 and increased protein and mRNA levels of p-AKT and p-PI3K in EJ and UMUC3 cells (Figures 6A–D); transfection of OE-ID2 resulted in a significant increase in ID2 levels and decreased protein levels of p-AKT and p-PI3K in cells (Figures 6A–D). Through the above experiments, we demonstrated that ID2 may be involved in BCa progression through PI3K/AKT signaling pathway.

## Discussion

In this study, we first examined the expression and prognostic value of four ID proteins in the TCGA BCa database. We found that ID2 was lowly expressed in BCa tumor tissues, ID2 expression correlated with TNM stage, grade and pathological stage, and that high ID2 expression was positively correlated with OS, DSS, and PFI. *In vivo* and *in vitro* functional experiments, we found that knockdown of ID2 promoted proliferation, migration, invasion and metastasis and inhibited apoptosis of BCa cells, while overexpression of ID2 significantly inhibited cell proliferation, migration, invasion and metastasis and promoted apoptosis. Mechanistically, we demonstrated that ID2 can be involved in BCa progression and metastasis through the PI3K/AKT signaling pathway.

Abnormal expression of many molecules or aberrant activation of signaling pathways can lead to the development, invasion and metastasis of BCa. Among them, PI3K/AKT signaling pathway is an important signaling pathway that is widely present in cells and can regulate cell growth, proliferation, apoptosis, metabolism, tumor invasion, and metastasis (Petrulea et al., 2015). This signaling pathway is abnormally activated in some BCa patients and is associated with the occurrence, development and prognosis of BCa (Sathe and Nawroth, 2018).

ID family proteins are members of the bHLH transcription factor family, and their negative regulatory role in the activation of bHLH transcription factors, which can inhibit cell differentiation by suppressing the binding of bHLH transcription factors to DNA and other tissue-specific bHLH (Kee, 2009). ID family proteins include ID1, ID2, ID3, and ID4, which are involved in the regulation of cell growth processes, including cell growth, differentiation, and death (Norton, 2000). ID family genes have been shown to be extensively involved in the development of a variety of cells and tissues in the body and have been associated with cancer (Ruzinova and Benezra, 2003; Wang and Baker, 2015).

Aberrant expression levels of ID2 protein have been reported in a variety of cancers, such as non-small cell lung cancer, breast cancer, esophageal squamous cell carcinoma (ESCC), and colorectal cancer (Yuen et al., 2007; Gray et al., 2008; Rollin et al., 2009; Liu et al., 2019). Yuen et al. (2007) found that ID1 and ID2 are highly expressed in ESCC and are markers of metastasis and prognosis in ESCC. Stighall et al. (2005) found that high ID2 protein expression was associated with a good prognosis in patients with primary breast cancer and reduced the invasiveness of breast cancer cells. In addition, Gray et al. (2008) found that ID2 protein was highly expressed in colorectal cancer samples and that intraperitoneal injection of ID2 small interfering RNA reduced the growth of colorectal cancer in mouse liver, suggesting that ID2 is a potential drug target for tumor therapy. A study of ID2 in bladder cancer found that ID2 was highly expressed in BCa and regulated by H19 (Luo et al., 2013). We speculate that there may be two reasons for the different findings in the present study and the study by Luo et al. (2013): first, it may be related to the tumor heterogeneity of bladder cancer (Chen et al., 2015; Warrick et al., 2019); second, patients who received preoperative chemotherapy or radiotherapy were excluded from the present study, and there were no relevant exclusion criteria for sample collection in the study by Luo et al. (2013). It has been found that treatment of human lung cancer NCI-H460 cells with an external drug (curcumin) can lead to high expression of ID1, ID2, and ID3 (Chiang et al., 2015). In the present study, we demonstrated that ID2 was significantly downregulated in BCa tissues at the mRNA level and protein level through the TCGA BCa database and clinical data from our center, high ID2 expression was negatively associated with tumor stage and positively correlated with overall survival, DSS and PFI. Furthermore, ID2 mechanistically inhibits BCa progression and metastasis through the PI3K/AKT signaling pathway.

## Conclusion

We found that ID2 expression was downregulated in BCa tissues and cell lines, and that low ID2 expression was associated with poor prognosis. ID2 could be a novel biomarker for BCa, and this signaling axis could be a potential therapeutic target for BCa.

## Data Availability Statement

The datasets presented in this study can be found in online repositories. The names of the repository/repositories and accession number(s) can be found in the article/Supplementary Material.

## Ethics Statement

This study was approved by the Ethics Committee of Shanghai Tenth People’s Hospital of Tongji University (SHSY-IEC-4.1/19-120/01). The patients/participants provided their written informed consent to participate in this study. Written informed consent was obtained from the individual(s) for the publication of any potentially identifiable images or data included in this article.

## Author Contributions

WM, MC, JG, and ML designed the research. WM, KW, and SS performed the research, wrote the manuscript, and analyzed the results. WM, JW, MC, JG, and ML edited the manuscript and provided critical comments. All authors read and approved the final manuscript.

## Conflict of Interest

The authors declare that the research was conducted in the absence of any commercial or financial relationships that could be construed as a potential conflict of interest.

## Publisher’s Note

All claims expressed in this article are solely those of the authors and do not necessarily represent those of their affiliated organizations, or those of the publisher, the editors and the reviewers. Any product that may be evaluated in this article, or claim that may be made by its manufacturer, is not guaranteed or endorsed by the publisher.

## Supplementary Material

Supplementary Figure 1 Expression of ID1, ID3, and ID4 in the TCGA database. **(A)** ID1 expression profile across all tumor samples and paired normal tissues. **(B)** The difference expression of ID1 in BCa tissues and adjacent normal tissues. **(C)** The difference expression of ID1 in BCa tissues and paired normal tissues. **(D)** The difference expression of ID1 in normal tissues of GTEx combined with TCGA and BCa tissues of TCGA. (**E–G**) Overall survival **(E)**, disease-specific survival **(F)**, and progress free interval **(G)** curve of BCa patients with low (*n* = 207) and high (*n* = 207) ID1 expression. **(H)** ID3 expression profile across all tumor samples and paired normal tissues. **(I)** The difference expression of ID3 in BCa tissues and adjacent normal tissues. **(J)** The difference expression of ID3 in BCa tissues and paired normal tissues. **(K)** The difference expression of ID3 in normal tissues of GTEx combined with TCGA and BCa tissues of TCGA. **(L–N)** Overall survival **(L)**, disease-specific survival **(M)** and progress free interval **(N)** curve of BCa patients with low (*n* = 207) and high (*n* = 207) ID3 expression. **(O)** ID4 expression profile across all tumor samples and paired normal tissues. **(P)** The difference expression of ID4 in BCa tissues and adjacent normal tissues. **(Q)** The difference expression of ID4 in BCa tissues and paired normal tissues. **(R)** The difference expression of ID4 in normal tissues of GTEx combined with TCGA and BCa tissues of TCGA. **(S–U)** Overall survival **(S)**, disease-specific survival **(T)**, and progress free interval **(U)** curve of BCa patients with low (*n* = 207) and high (*n* = 207) ID4 expression.

Supplementary Table 1 Antibodies list.

Supplementary Table 2 RNA sequencing data.

Supplementary Table 3 GO analysis and KEGG analysis results.
