# Supplementary figures and images for: ID2 Inhibits Bladder Cancer Progression and Metastasis via PI3K/AKT Signaling Pathway
Source: Front Cell Dev Biol. 2021 Oct 22;9:738364. doi: 10.3389/fcell.2021.738364 (PMC8570141; doi:10.3389/fcell.2021.738364)

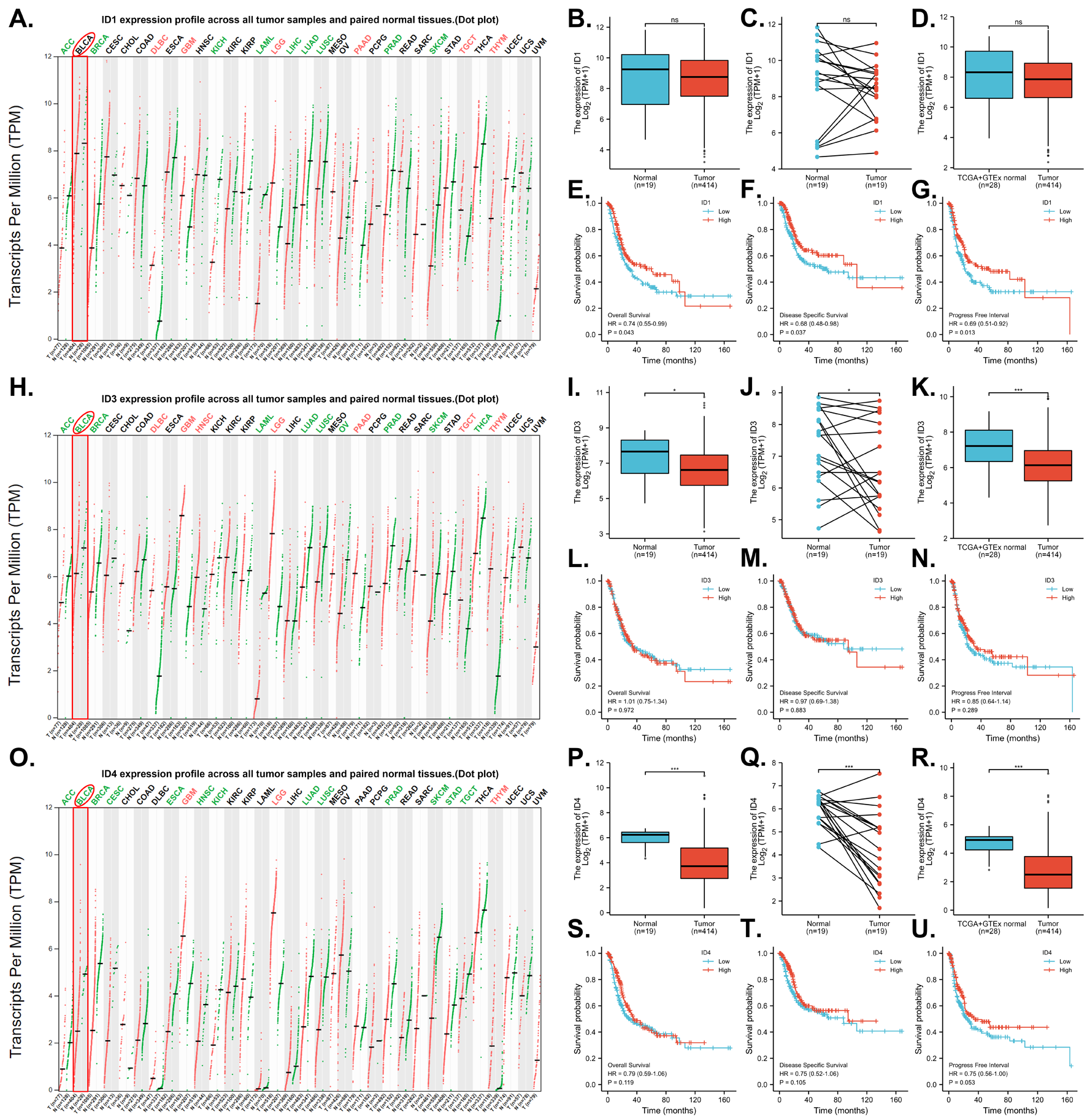

Supplement: Supplementary Figure 1 — Expression of ID1, ID3, and ID4 in the TCGA database. (A) ID1 expression profile across all tumor samples and paired normal tissues. (B) The difference expression of ID1 in BCa tissues and adjacent normal tissues. (C) The difference expression of ID1 in BCa tissues and paired normal tissues. (D) The difference expression of ID1 in normal tissues of GTEx combined with TCGA and BCa tissues of TCGA. (E–G) Overall survival (E), disease-specific survival (F), and progress free interval (G) curve of BCa patients with low (n = 207) and high (n = 207) ID1 expression. (H) ID3 expression profile across all tumor samples and paired normal tissues. (I) The difference expression of ID3 in BCa tissues and adjacent normal tissues. (J) The difference expression of ID3 in BCa tissues and paired normal tissues. (K) The difference expression of ID3 in normal tissues of GTEx combined with TCGA and BCa tissues of TCGA. (L–N) Overall survival (L), disease-specific survival (M) and progress free interval (N) curve of BCa patients with low (n = 207) and high (n = 207) ID3 expression. (O) ID4 expression profile across all tumor samples and paired normal tissues. (P) The difference expression of ID4 in BCa tissues and adjacent normal tissues. (Q) The difference expression of ID4 in BCa tissues and paired normal tissues. (R) The difference expression of ID4 in normal tissues of GTEx combined with TCGA and BCa tissues of TCGA. (S–U) Overall survival (S), disease-specific survival (T), and progress free interval (U) curve of BCa patients with low (n = 207) and high (n = 207) ID4 expression. [file Image_1.TIF]

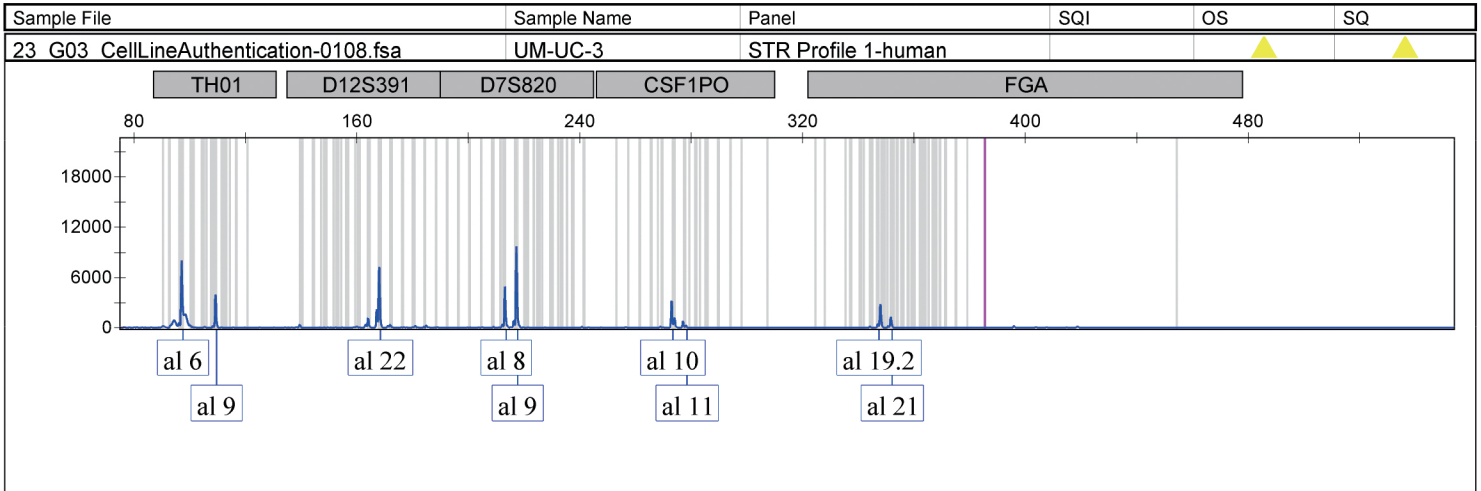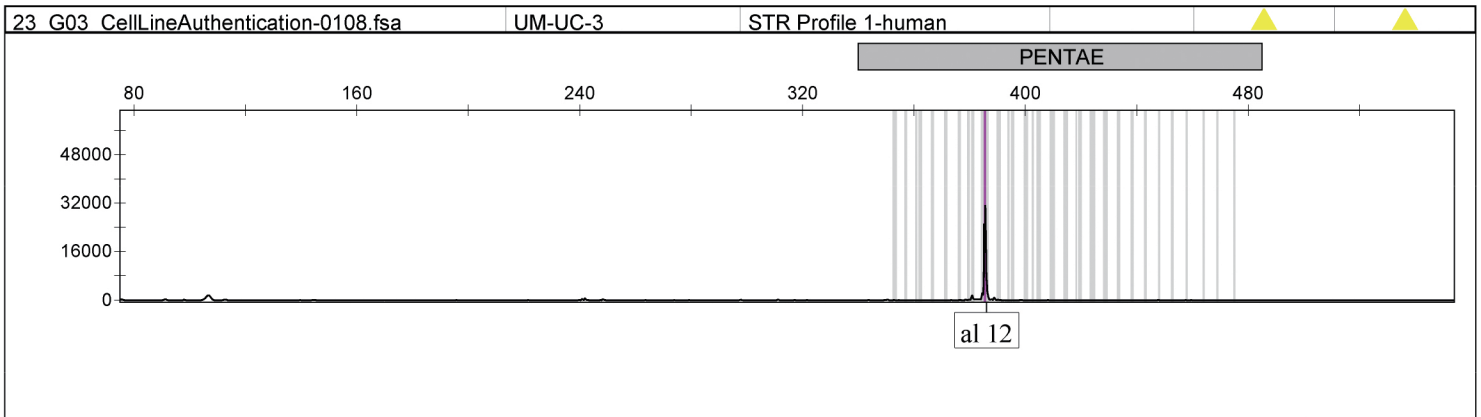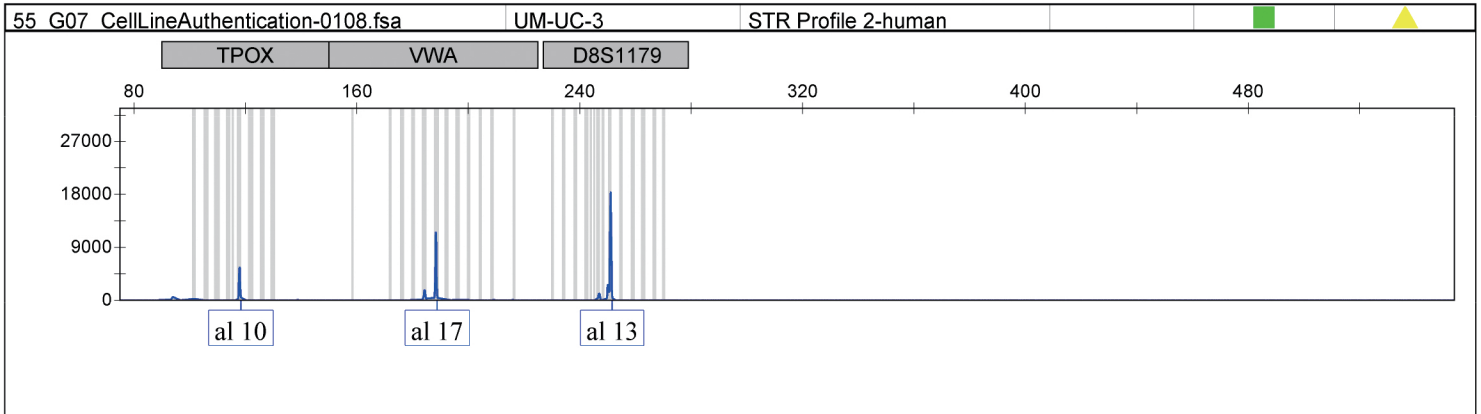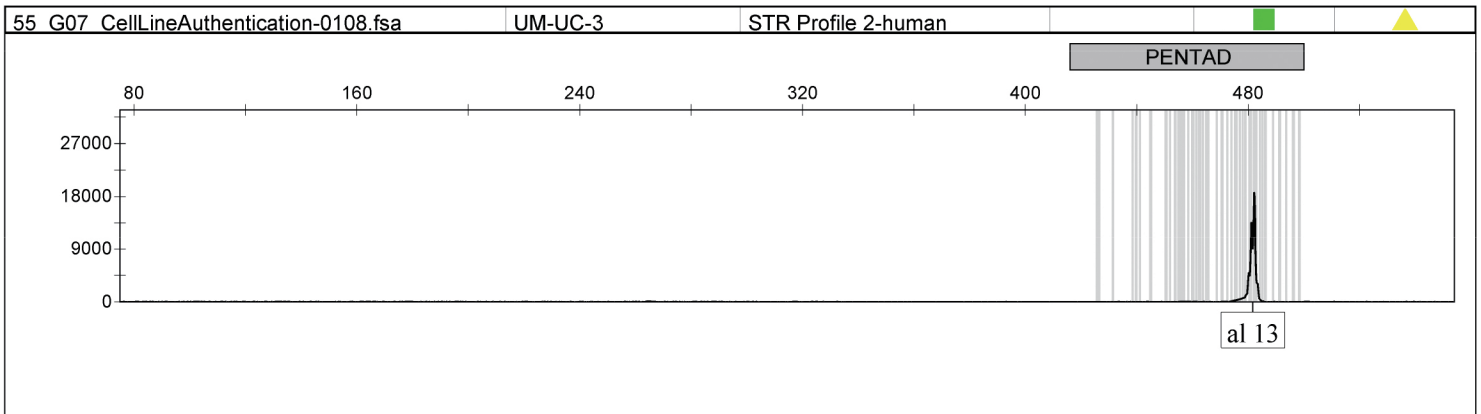

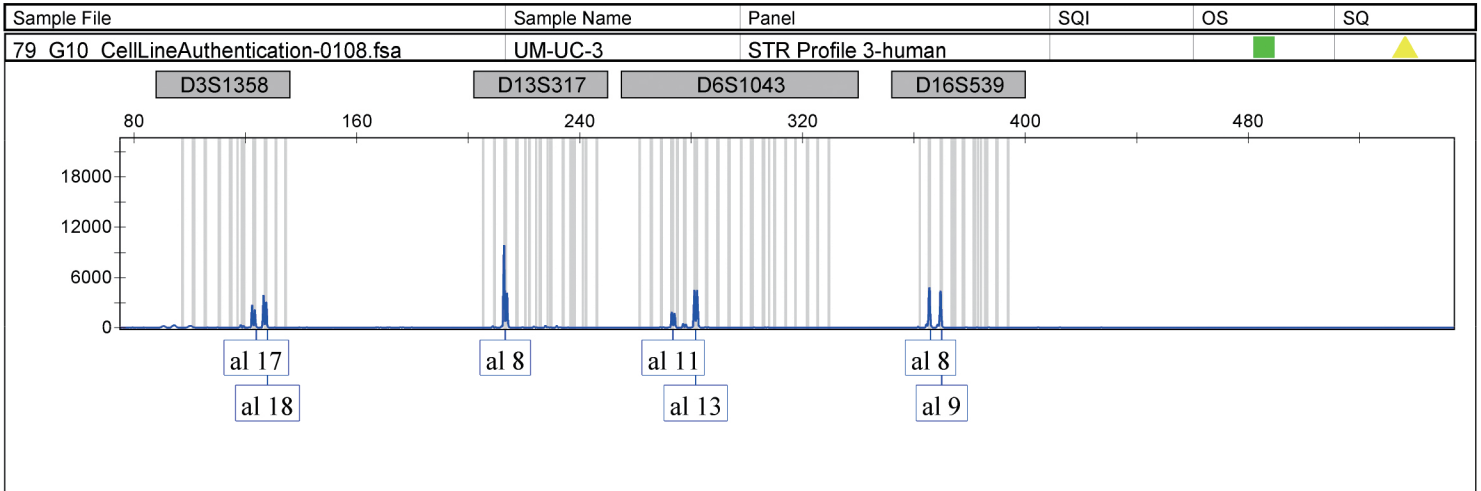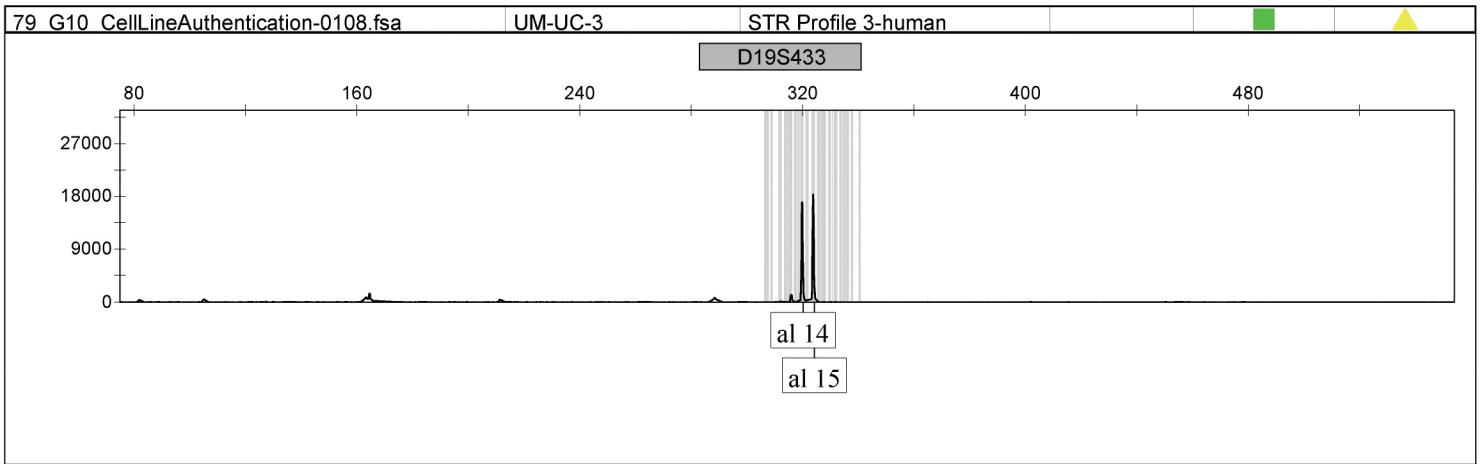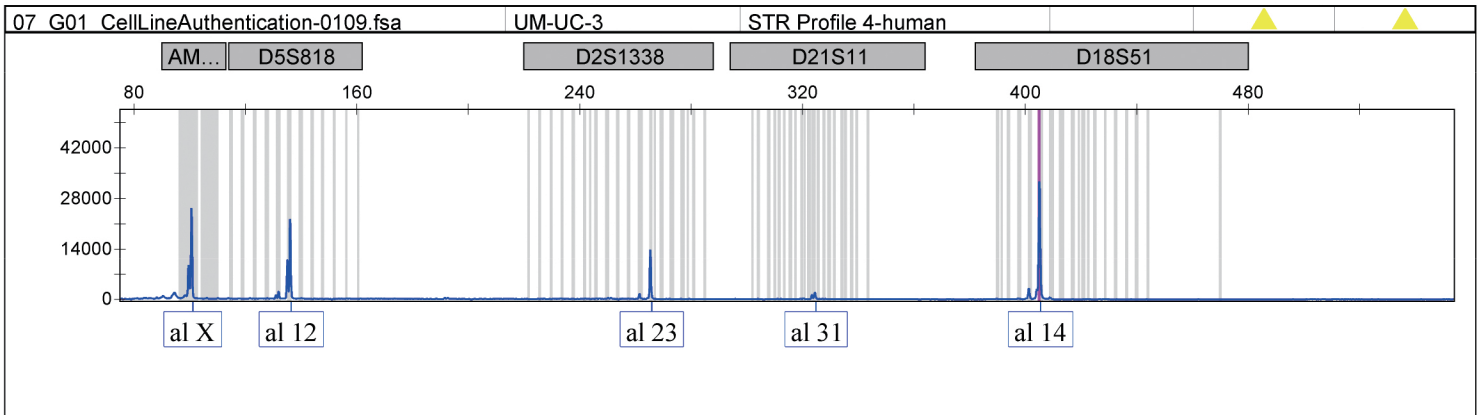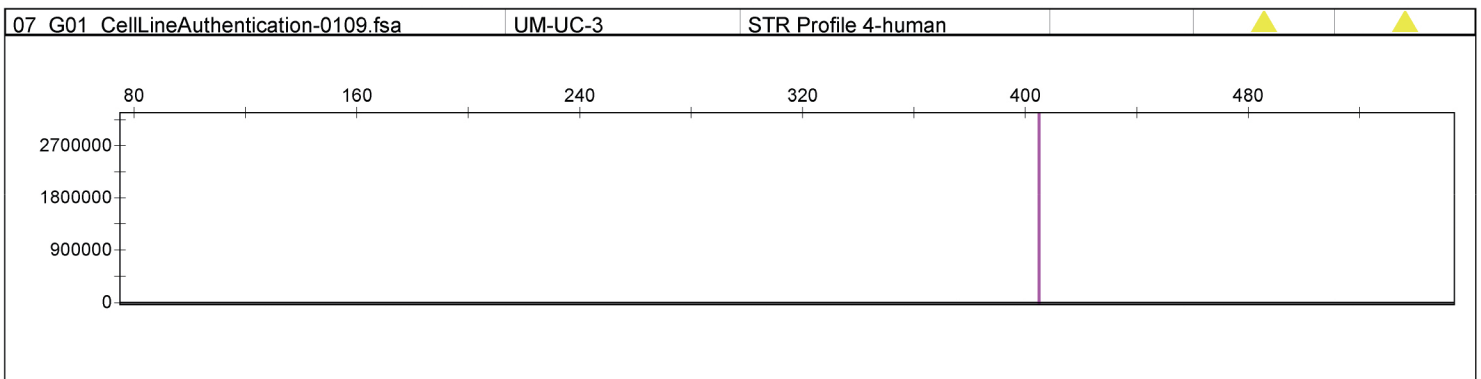

Supplement: Supplementary file 6 [file Data_Sheet_2.PDF]
