# Supplementary material for: ID2 Inhibits Bladder Cancer Progression and Metastasis via PI3K/AKT Signaling Pathway
Source: Front Cell Dev Biol. 2021 Oct 22;9:738364. doi: 10.3389/fcell.2021.738364 (PMC8570141; doi:10.3389/fcell.2021.738364)
Supplement: Supplementary Table 2 — RNA sequencing data. [file Table_1.docx]

**Table S1.** Antibodies list.

| **Name** | **Company** | **Number** |
| --- | --- | --- |
| p-PI3K | Cell Signaling Technology | 17366S |
| PI3K | Cell Signaling Technology | 4257S |
| p-AKT | Abcam | ab81283 |
| AKT | Abcam | ab18785 |
| ID2 | Absin | Abs135698 |
| GAPDH | Abcam | ab8245 |
| Goat Anti-Rabbit IgG H&L (HRP) | Abcam | ab6721 |
| Goat Anti-Mouse IgG H&L (HRP) | Abcam | ab6789 |
|  |  |  |
