# Supplementary material for: ID2 Inhibits Bladder Cancer Progression and Metastasis via PI3K/AKT Signaling Pathway
Source: Front Cell Dev Biol. 2021 Oct 22;9:738364. doi: 10.3389/fcell.2021.738364 (PMC8570141; doi:10.3389/fcell.2021.738364)

Applied Biosystems
GeneMapper 4.0

CellLineAuthentication-1122

Sample File	Sample Name	Panel	SQI	OS	SQ
80_H10_CellLineAuthentication-1-1127.FSA	EJ	STR Profile 1-human			

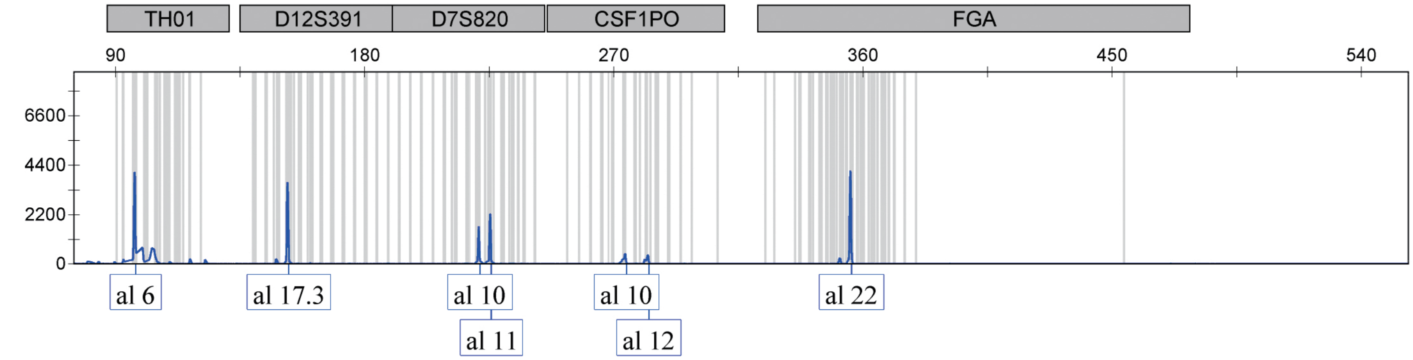

Sample File	Sample Name	Panel	SQI	OS	SQ
80_H10_CellLineAuthentication-1-1127.FSA	EJ	STR Profile 1-human			

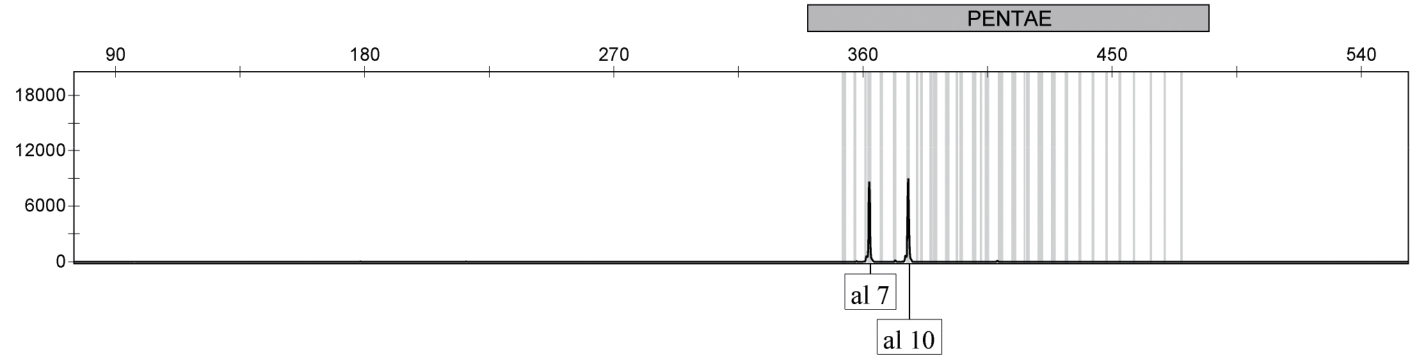

Sample File	Sample Name	Panel	SQI	OS	SQ
08_H01_CellLineAuthentication-2-1127.FSA	EJ	STR Profile 2-human			

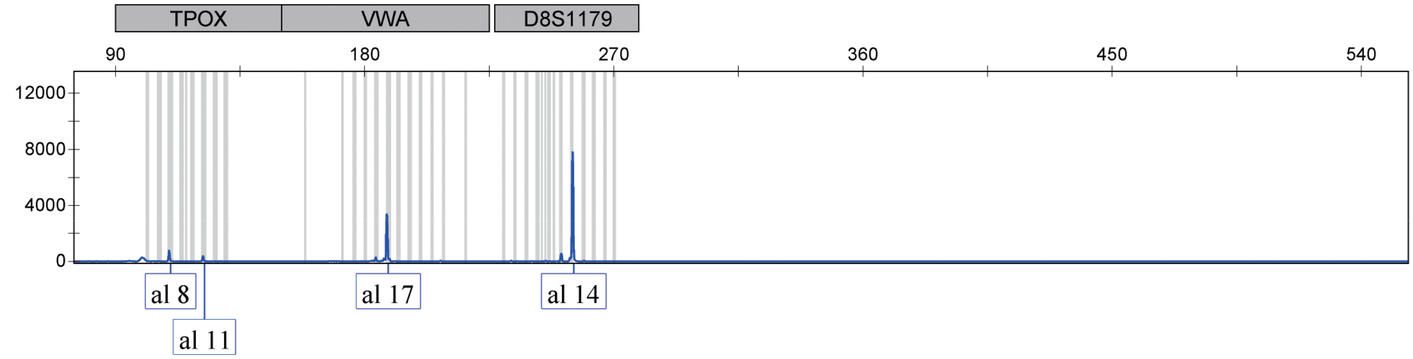

Sample File	Sample Name	Panel	SQI	OS	SQ
08_H01_CellLineAuthentication-2-1127.FSA	EJ	STR Profile 2-human			

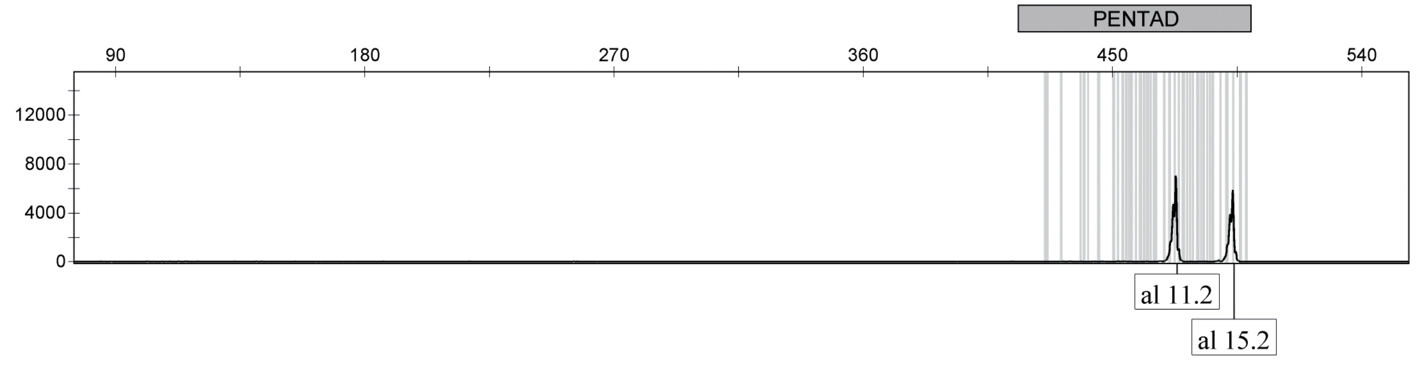

Sample File	Sample Name	Panel	SQI	OS	SQ
48_H06_CellLineAuthentication-2-1127.FSA	EJ	STR Profile 3-human			

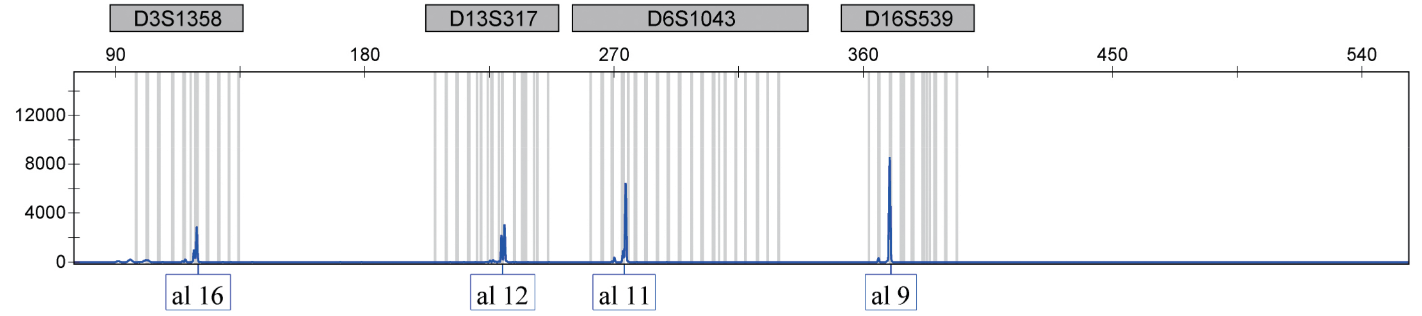

Sample File	Sample Name	Panel	SQI	OS	SQ
48_H06_CellLineAuthentication-2-1127.FSA	EJ	STR Profile 3-human			

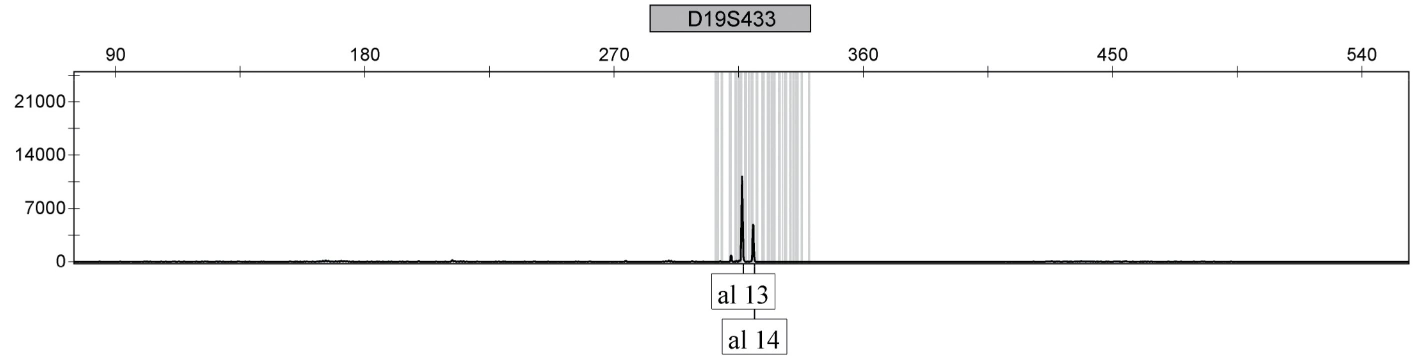

Applied Biosystems
GeneMapper 4.0

CellLineAuthentication-1122

Sample File	Sample Name	Panel	SQI	OS	SQ
08_H01_XiBaoJianDing1122Pi-2(1-5)-1129.FSA	EJ	STR Profile 4-human			

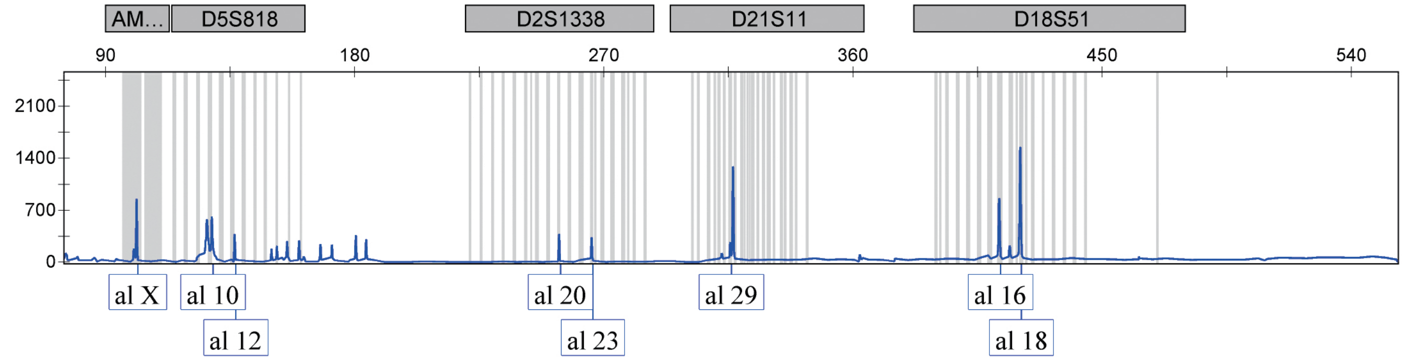

Sample File	Sample Name	Panel	SQI	OS	SQ
08_H01_XiBaoJianDing1122Pi-2(1-5)-1129.FSA	EJ	STR Profile 4-human			

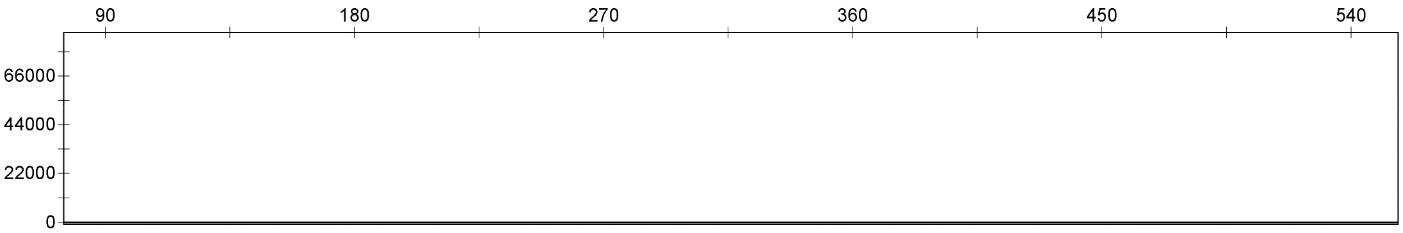

Supplement: Supplementary file 5 [file Data_Sheet_1.PDF]
